# Genotype-Environment Correlation in the Era of DNA

**DOI:** 10.1007/s10519-014-9673-7

**Published:** 2014-09-07

**Authors:** Robert Plomin

**Affiliations:** MRC Social, Genetic and Developmental Psychiatry Centre, Institute of Psychiatry, King’s College London, PO80, De Crespigny Park, London, SE5 8AF UK

**Keywords:** Genotype-environment correlation, Genome-wide complex trait analysis (GCTA), Socioeconomic status, Group-level environments, Active model of experience

## Abstract

One of John Loehlin’s many contributions to the field of behavioral genetics involves gene-environment (GE) correlation. The empirical base for GE correlation was research showing that environmental measures are nearly as heritable as behavioral measures and that genetic factors mediate correlations between environment and behavior. Attempts to identify genes responsible for these phenomena will come up against the ‘missing heritability’ problem that plagues DNA research on complex traits throughout the life sciences. However, DNA can also be used for quantitative genetic analyses of unrelated individuals (Genome-wide Complex Trait Analysis, GCTA) to investigate genetic influence on environmental measures and their behavioral correlates. A novel feature of GCTA is that it enables genetic analysis of family-level environments (e.g., parental socioeconomic status) and school-level environments (e.g., teaching quality) that cannot be investigated using within-family designs such as the twin method. An important implication of GE correlation is its shift from a passive model of the environment imposed on individuals to an active model in which individuals actively create their own experiences in part on the basis of their genetic propensities.

## Introduction

John Loehlin’s influence on my career involves gene-environment (GE) correlations of a personal as well as scientific kind. At the personal level, he introduced me to behavioral genetics in 1971 when I was a second-year graduate student in psychology at the University of Texas at Austin. He contributed to a ‘core course’ on behavioral genetics, which included the first *Annual Review of Psychology* chapter on behavioral genetics (Lindzey et al. 1971) and was compulsory for all psychology graduate students. For GE correlation reasons that involve appetite more than aptitude, this course, and especially John Loehlin’s contribution, made me realize that behavioral genetics was the field for me, even though none of the other 40 students in the core course were similarly enticed to behavioral genetics.

The beauty and clarity of John Loehlin’s writing also attracted me to behavioral genetics. It cannot be a coincidence that his undergraduate degree was English and that he is passionate about poetry. In part because of his writing and the clear thinking that underlies it, his books form part of the bedrock of behavioral genetics, bringing lucidity to difficult topics such as race differences (Loehlin et al. 1975), personality (Loehlin 1992; Loehlin and Nichols 1976), and latent variable models (Loehlin 1987). My favorite is his 1976 book on personality, *Heredity, environment, and personality: A study of 850 sets of twins.* Three quotes from this book illustrate the clarity and lack of pomposity in his writing—as well as the importance of his findings:

- *The first clear statement about the importance of non*-*shared environment:*“As far as personality and interests are concerned, then, it would appear that the relevant environments of a pair of twins are no more alike than those of two members of the population paired at random. Can this possibly be true? (p. 91)… Thus, a consistent – though perplexing – pattern is emerging from the data (and it is not purely idiosyncratic to our study). Environment carries substantial weight in determining personality – it appears to account for at least half the variance – but that environment is one for which twin pairs are correlated close to zero… In short, in the personality domain we seem to see environmental effects that operate almost randomly with respect to the sorts of variables that psychologists (and other people) have traditionally deemed important in personality development. What can be going on?” (p. 92).
- *Nearly all psychological traits show moderate genetic influence (lack of differential heritability):* “Its message might roughly be translated: ‘Identical twins correlate about .20 higher than fraternal twins, give or take some sampling fluctuation, and it doesn’t much matter what you measure – whether the difference is between .75 and .55 on ability measures, between .50 and .30 on a personality scale, or between .35 and .15 on a self-concept composite” (p. 35).
- *One of the earliest multivariate genetic analyses using twin data:* “The motivation underlying such analyses is the hope that they may provide a powerful tool for studying how genetic and environmental influences affect phenotypic traits. The basic reasoning runs something like this: It is unlikely that our convenient phenotypic trait measures are aligned in a simple one-to-one fashion with either the genetic or the environmental sources of influence upon them. If they are not, the effects of such influences should often show up more clearly on the associations among traits than on the measures of the individual traits themselves. Thus, two genetically independent traits might be correlated because they are subject to common environmental influences, or two traits that share no important environmental inputs might both be affected by a particular gene or genes (‘pleiotropy’)” (p. 75).

More than 30 years later, his work continues to advance these topics of nonshared environment (Loehlin 2007; Loehlin and Martin 2011b); differential heritability for personality traits (Loehlin 2012); and multivariate genetic issues especially in relation to a general factor of personality (Loehlin 2011; Loehlin and Horn 2012; Loehlin and Martin 2011a, 2013). He has also written about GE correlation and other aspects of the interplay between genes and environment (Loehlin 2010a, b). The beauty of his writing continues to shine through his most recent papers (e.g., Loehlin 2013).

John Loehlin was also responsible for launching my career in a very practical way by recommending me for an Assistant Professor position that suddenly materialized at the Institute for Behavioral Genetics as I was finishing my dissertation. His influence on my career did not decrease with the 1,000 miles between Austin and Boulder. I was so impressed with the Texas Adoption Study that John Loehlin and Joseph Horn had established while I was a graduate student at Texas (Horn et al. 1979; Horn and Loehlin 2010; Loehlin et al. 1981) that I decided, with John DeFries, to conduct a study of newborn adoptees in Colorado, which became the Colorado Adoption Project (Plomin and DeFries 1985).

Another example of John Loehlin’s impact on my scientific career is my interest in GE correlation, which was sparked by John Loehlin while I was at Texas. This interest led to a paper with John Loehlin and John DeFries on GE correlation and interaction, which continues to be my most highly cited paper (Plomin et al. 1977). In that paper, the best writing was John Loehlin’s, including the concluding paragraph, which I quote here because it is about the interpretation of GE correlation. Roberts (1967) had argued that GE correlation is ‘really’ genetic and that “it matters not one whit whether the effects of the genes are mediated through the external environment or directly through, say, the ribosomes” (p. 218). We argued that GE correlation is ‘really’ a correlation between genes and environment, and John Loehlin wrote:

“Although formally it may not matter one whit in which way the effects of the genes are mediated, in practice it often matters quite a few whits, especially if one should happen to be interested in intervening in the process. Changing behavior by changing parental attitudes is a decidedly different proposition from tinkering with the ribosomes, even though a similar behavioral change might conceivably be brought about by either means” (p. 321).

The wit of ‘whits’ and ‘tinkering with the ribosomes’ are good examples of the freshness and vividness of his writing—in addition to making a critical point. He also wrote the last sentence of the paper: “And one day, perhaps, we may yet get to the ribosomes” (p. 321).

### GE correlation

In our 1977 paper, we considered the effects of GE correlation and interaction on quantitative genetic estimates, proposed three types of GE correlation (passive, reactive and active), and suggested ways to assess GE correlation and interaction. In the present paper, I will briefly summarize research on GE correlation, highlighting new developments using DNA.

GE correlation can be viewed as genetic influence on exposure to environments—literally, a correlation between genetic propensities and experiences. In contrast, GE interaction denotes genetic influence on response to environments, that is, a conditional relationship in which the effect of the environment on a phenotype depends on genotype (Kendler and Eaves 1986). In other words, GE correlation refers to genetic mediation of associations between environments and traits, whereas GE interaction involves genetic moderation of these associations. For example, much research in the past decade investigates moderation of environment-trait correlations by candidate genes, following one of the most highly cited papers in the behavioral sciences reporting that the influence of life stress on depression depends on DNA variation in a serotonin transporter (Caspi et al. 2003). GE interaction and GE correlation assume different models of the environment. The GE interaction model assumes an environment ‘out there’ that is imposed on the individual to which the individual reacts in part on the basis of genetic propensities. The essence of active GE correlation is choice: Individuals select, modify and create experiences that are correlated with their genetic propensities. Although there is much to learn about GE interaction (Petrill et al. 2013), I suggest that active GE correlation will ultimately be more enlightening about the developmental interplay in which genotypes use the environment—from cells to society—to develop into phenotypes.

GE correlation is responsible for one of the most extraordinary findings in behavioral genetics: environmental measures used widely in the behavioral sciences show nearly as much genetic influence as behavioral measures (Plomin and Bergeman 1991). By 1991, this was shown in 18 studies. In 1992, John Loehlin wrote that “the complexities of GE correlation represent a research area which has barely been touched empirically” (Loehlin 1992, p. 126). Now there are more than 100 empirical reports that explore a wide range of environmental measures such as life events, social support, parenting and even children’s television viewing. One review of 55 independent studies analyzing environmental measures as dependent variables in genetically sensitive designs found an average heritability of 27 % across 35 different environmental measures (Kendler and Baker 2007). A recent review of 32 studies on parenting in child-centered designs (i.e., where twins are children) reported an average heritability of 23 % (Avinun and Knafo 2013).

If there is genetic influence on environmental measures as well as behavioral measures, it is possible that associations between environmental measures and behavioral measures are mediated genetically. Most GE correlation research in the past decade has moved beyond merely demonstrating genetic influence on environmental measures, to using multivariate genetic analysis to assess genetic mediation on associations between environment and behavior (Plomin 1994).

Scores of studies show that genetic factors often significantly mediate associations between environmental and behavioral measures, such as correlations between family environment and the development of children’s psychopathology (Knafo and Jaffee 2013). These findings indicate that such correlations cannot safely be interpreted causally as the effect of environment on behavior. They also indicate the extent to which such correlations are truly environmental in origin. For example, a recent study showed that, despite some genetic influence on household chaos, its effect on subsequent disruptive behavior was environmentally mediated (Jaffee et al. 2012). In the search for such true environmental effects, it is important to disentangle passive, reactive and active types of GE correlation, and John Loehlin has contributed models that can do this (e.g., Loehlin and DeFries 1987; Loehlin 2010b; Plomin et al. 1985). A powerful design to disentangle types of GE correlation and to identify true environmental effects is the children of twins design (D’Onofrio et al. 2003) and the extended children of twins design (Narusyte et al. 2008).

## Within-family versus between-family environmental factors

Most GE correlation research uses the twin design that compares resemblance within pairs of monozygotic and dizygotic twins. For GE correlation, this limits the twin design to investigating experiences that differ for a pair of twins growing up in the same family, living in the same neighborhood, and attending the same school. This is an important limitation because many crucial environmental variables are the same for two children in a family (e.g., parental SES, chaos in the home), in a neighborhood (e.g., crime and safety, green space), and in a school (e.g., school infrastructure such as resources and teaching quality, school composition such as demographic characteristics). Because these environmental variables are the same for members of a twin pair, they would be read as shared environmental influences in a twin design. However, the correlation between family-level environmental factors such as parental SES and children’s developmental outcomes could be mediated genetically but the twin design would not ‘see’ it. This is a problem primarily for research on children, but also for research that attempts to study the childhood origins of adult behavior. It is much less of a problem for twin studies investigating the effects of contemporaneous environments of adults to the extent that members of adult twin pairs live separate lives.

One way to circumvent this within-family limitation of the twin design is to recast between-family factors, such as family chaos, as individual differences in children’s perceptions of their family chaos. For example, we have studied GE correlation using children’s perceptions of their experiences at home (e.g., Hanscombe et al. 2010, 2011) and school (e.g., Asbury et al. 2008; Haworth et al. 2013). Young people (twins) in the same family and same school report differences in their perceptions, and these self-reported perceptions show genetic influence, but they only correlate modestly with educational outcomes. In retrospect, this research evokes the allegory of losing one’s wallet (GE correlation) in the dark alley (family-level, neighborhood-level, and school-level environments) but looking for it under the streetlamp (individual-level perceptions) because the light is better.

The parent-offspring adoption design can address family-level environmental factors, for example, by comparing the correlation between family environment and children’s development in non-adoptive and adoptive homes (Loehlin and DeFries 1987). However, because it is increasingly difficult to conduct adoption studies, twin studies will continue to be most widely used and will miss most of the environmental action, which is between families, and thus shortchange research on GE correlation.

Research on GE correlation in general—and family-level, neighborhood-level, and school-level environments in particular—will be revolutionized by a new quantitative genetic technique that uses DNA alone in samples of unrelated individuals rather than twins or adoptees.

## Genome-wide complex trait analysis (GCTA)

If heritable factors contribute to individual differences as assessed by environmental measures, this means that DNA differences are responsible for these effects. Nothing would advance GE correlation research more than identifying some of these DNA differences. Candidate gene association studies of environmental measures began to be reported as early as 2006 (Lucht et al. 2006) and the first genome-wide association study of an environmental measure was reported in 2008 (Butcher and Plomin 2008). However, this research has run up against the problem that plagues research on complex traits throughout the life sciences: missing heritability, which refers to the wide gap between heritability and the variance explained by identified DNA associations (Plomin and Simpson 2013). Genome-wide association studies throughout the life sciences have shown that there are no DNA associations of large effect size. The largest effect sizes are less than 1 % and the smallest effect sizes are likely to be infinitesimal. Very large samples will be needed to detect such small effects.

An unforeseen benefit of genome-wide association studies is that the data required for them—large samples of unrelated individuals, each genotyped for hundreds of thousands of single-nucleotide polymorphisms (SNPs)—can be used for quantitative genetic analyses of genetic influence. This technique, often called *Genome-wide Complex Trait Analysis (GCTA)*, is the first new human quantitative genetic technique in a century (Yang et al. 2010, 2011b, 2013a). The significance of GCTA is that it can estimate the net effect of genetic influence using DNA of unrelated individuals rather than using familial resemblance in groups of special family members who differ in genetic relatedness such as twins and adoptees (Zaitlen and Kraft 2012).

Unlike genome-wide association, GCTA does not identify specific SNPs associated with a trait. Like other quantitative genetic designs such as the twin design, GCTA uses genetic similarity to predict phenotypic similarity. However, instead of using genetic similarity from groups differing by a known degree of genetic similarity, such as MZ and DZ twins, GCTA uses genetic similarity (Genetic Relatedness Matrix) for each pair of unrelated individuals based on that pair’s overall similarity across hundreds of thousands of SNPs; each pair’s genetic similarity is then used to predict their phenotypic similarity. Even remotely related pairs of individuals are excluded so that chance genetic similarity is used as a random effect in a mixed linear maximum likelihood model to decompose phenotypic variance into genetic variance as captured by the additive effects of causal variants in linkage disequilibrium with SNPs genotyped on DNA arrays (Yang et al. 2011b). The power of the method comes from comparing, not just two groups like MZ and DZ twins, but millions of pairs of individuals. For example, a sample of 6,000 individuals provides eight million pair-by-pair comparisons. In contrast to the twin design, which only requires a few hundred pairs of twins to estimate moderate heritability and does not need DNA, GCTA requires samples of thousands of individuals because the method attempts to extract a small signal of genetic similarity from the massive noise of hundreds of thousands of SNPs. A handy GCTA power calculator is available (http://spark.rstudio.com/ctgg/gctaPower/). For example, a sample of 6,000 has 80 % power to detect a GCTA heritability estimate of 15 %. However, power declines sharply with smaller sample sizes: Samples of 4,000 and 2,000 have 80 % power to detect GCTA heritability estimates of 22 and 45 %, respectively. As discussed later, GCTA heritability estimates are limited to detecting additive effects of the common SNPs on current GWA microarrays, which results in GCTA heritability estimates often being about half of twin study estimates. GCTA can also be used to estimate genetic influence within pairs of siblings (Visscher et al. 2006; Hemani et al. 2013). Because siblings vary in genetic relatedness around their average genetic relatedness of 50 %, GCTA-estimated differences within pairs of siblings can be used in an analogous way to explain phenotypic differences within the sibling pairs. A benefit of this within-family design is that it controls for between-family stratification; a disadvantage in the present context, which is discussed later, is that it is limited to measures that vary within families. More generally, because much larger samples are needed to apply GCTA within sibling pairs, GCTA will primarily be applied to unrelated individuals.

GCTA has been used in scores of studies to estimate genetic influence for physical traits such as height and weight (Yang et al. 2010, 2011a), physiological traits (Yang et al. 2013b), medical disorders (Keller et al. 2012; Lee et al. 2013), psychiatric disorders (Cross-Disorder Group of the Psychiatric Genomics Consortium 2013; Lee et al. 2011, 2012a; Lubke et al. 2012), alcohol dependence (Kos et al. 2013), pharmacogenetics (Tansey et al. 2013; Verweij et al. 2013; Vrieze et al. 2013), personality (McGue et al. 2013; Rietveld et al. 2013a; Vinkhuyzen et al. 2012), behavioral economics (Benjamin et al. 2012; van der Loos et al. 2013), and cognitive abilities (Benyamin et al. 2013; Davies et al. 2011; Deary et al. 2012; Plomin et al. 2013). GCTA has recently been extended to bivariate analyses (Lee et al. 2012b), which enables more sophisticated quantitative genetic analyses such as analyses of age-to-age change and continuity (Deary et al. 2012; Trzaskowski et al. 2013d) and multivariate analyses (Trzaskowski et al. 2013b, c). An important feature of bivariate GCTA analysis is that its estimates of genetic correlations are similar to twin study estimates even though GCTA estimates of genetic variance and covariance are about half the estimates from twin analyses (Trzaskowski et al. 2013d).

There are three benefits of GCTA analysis. First, GCTA makes it possible to conduct quantitative genetic analyses in any large sample with genome-wide genotypes. In this way, GCTA will make behavioral genetics available to a much larger community. Second, GCTA can be used to confirm the results of twin studies. Comparisons between GCTA and twin study estimates of heritability generally show that GCTA accounts for about half the heritability estimates in twin studies (Plomin et al. 2013), perhaps less for behavior problems and personality (Trzaskowski et al. 2013a).

A third benefit is that GCTA provides insight into genetic architecture and the missing heritability problem. GCTA only detects genetic effects tagged by the common SNPs (allele frequencies greater than 1 %) that have until recently been incorporated in commercially available DNA microarrays used in genome-wide association studies. In addition, GCTA is limited to detecting the additive effects of SNPs; it cannot detect gene–gene (or gene-environment) interaction. Thus, if GCTA heritability estimates are half the twin study heritability estimates, the additive effects of common SNPs can in theory account for about half of the heritability estimated from twin studies. The ‘missing GCTA heritability’, the gap between GCTA and twin study heritability estimates, could be due to nonadditive effects or the effects of rare DNA variants. In other words, GCTA estimates the lower limit of heritability from twin studies and the upper limit for genome-wide association studies. These generalizations may not apply equally to all behavioral domains. For example, childhood behavior problems and personality seem to show a greater gap between GCTA estimates and twin estimates than do other domains such as cognitive abilities; this may be due to greater assortative mating for cognitive abilities which increases additive genetic variance or to greater nonadditive effects for behavior problems and personality (Trzaskowski et al. 2013a).

## GCTA and GE correlation: group-level environments

GCTA can also be used to study GE correlation. In addition to investigating genetic influence on environmental measures (Power et al. 2013) and genetic mediation of associations between environment and behavior (Harlaar et al. 2014), GCTA can remedy the problem raised above concerning group-level environmental factors. That is, twin studies are limited to investigating within-family (twin-specific) experiences, whereas many important environmental factors are the same for two children in a family. Because GCTA is based on comparisons between unrelated individuals, the method focuses entirely on differences between families, in contrast to the twin method, which is a within-family analysis, comparing differences within pairs of twins in a family to differences between families. For this reason, we can use GCTA to investigate whether genetic factors contribute to family-level, neighborhood-level, and school-level environmental variables and their association with child outcomes.

It may seen counter-intuitive to look for genetic influence on such group-level environments, but children are not randomly assigned to families, neighborhoods, or schools—they are grouped genetically. Nuclear families are genetically defined groups, so average differences between families such as family SES can obviously be affected by genetic differences between families. But what about neighborhood-level and school-level environmental variables—how can ‘environmental’ differences between neighborhoods and between schools be genetic in origin? The answer is that genetic influence can emerge from all three types of GE correlation mentioned earlier. A group-level ‘passive’ GE correlation is possible because schools reflect families who live in those districts. Group-level ‘reactive’ GE correlation can be created by school intake policies such as selecting children on the basis of their performance on school entrance exams and interviews or on the basis of religious affiliation. Group-level ‘active’ GE correlation can occur, especially in secondary schools, when parents and pupils select schools that are correlated with the children’s abilities and interests.

As an empirical example of group-level genetics, we applied GCTA to genome-wide genotypes from 3,000 unrelated children to investigate family socio-economic status (SES), a composite of parental education and occupational status, and its association with children’s intelligence (Trzaskowski et al. 2014). Univariate GCTA indicated that phenotypic variance between families for SES is significantly due to genetic differences. The univariate GCTA heritability estimates for family SES were 0.18 when the children were age 2 and 0.19 when the children were age 7. It should be noted that genome-wide genotypes of one child per family were used to estimate genetic influence on family SES. Because the children’s genotypes only weakly reflect causal genetic factors responsible for their parents’ education and occupation, one might expect that parents’ DNA, not available in this study, would yield a higher GCTA heritability estimate of family SES because the family SES composite is constructed from the parents’ education and occupation. However, a similar GCTA heritability estimate of 0.22 (0.04 standard error, SE) has been reported for adult educational attainment based on the adults’ own DNA (Rietveld et al. 2013b). Another study also reported a similar GCTA heritability estimate of 0.19 (0.05 SE) for adult educational attainment as well as for an index of deprivation (0.21, 0.05 SE; Marioni et al. 2014). Bivariate GCTA yielded a genetic correlation of 0.83 (0.16 SE) between the adults’ own intelligence and their educational attainment, but the genetic correlation was much lower between their intelligence and the index of deprivation (0.16, 0.16 SE).

**Fig. 1:**
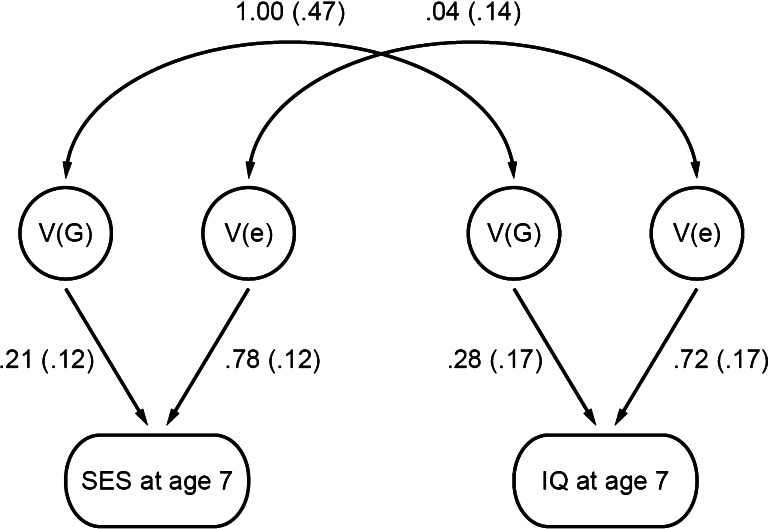
Bivariate GCTA showing genetic influence on family-level SES and on children’s IQ at age 7, and a genetic correlation of 1.0 between them. Although this path model looks like the result of a twin study, the within-family twin design cannot be used to analyze between-family environmental variables such as family-level SES; this path model describes GCTA results based on DNA from unrelated children. (Used with permission from Trzaskowski et al. 2014.)

A strength of the child-based design using children’s genotypes in GCTA analyses rather than genotypes of their parents is that it captures the genetic influence of family SES on the children themselves. This feature of the design facilitates a bivariate GCTA that assesses the extent to which the well-known correlation between family SES and cognitive development—about 0.30 in meta-analyses (Sirin 2005)—is mediated genetically. In the study described above (Trzaskowski et al. 2014), a GCTA genetic correlation near 1.0 emerged between family-level SES and children’s intelligence, as shown in Fig. 1. Moreover, genes almost entirely accounted for the phenotypic correlation of 0.30 between family SES and children’s intelligence. However, the large standard errors (shown in parentheses in Fig. 1), especially for the genetic correlation, indicate that samples larger than 3,000 are needed for definitive GCTA estimates.

## GE correlation and an active model of experience

GE correlation challenges current conceptions of the environment as something ‘out there’ that happens passively to children. Finding genetic influence on measures of the environment and on their association with outcomes will make us rethink how the environment works, leading to an active model of experience in which children select, modify and create environments correlated with their genetic propensities. This active model of experience supports an educational trend in the direction of personalized learning, making the educational environment fit the pupil’s profile of strengths and weaknesses—and appetites as well as aptitudes—rather than using a one-size-fits-all curriculum (Asbury and Plomin 2013).

John Loehlin has written about this active model of GE correlation in relation to the development of social attitudes, which highlights the importance of choice. It is fitting for this festschrift in honor of John Loehlin to let him have the last word, especially because the environment he provided was a crucial component in the GE correlations of my life:

“We may view this as a kind of cafeteria model of the acquisition of social attitudes. The individual does not inherit his ideas about fluoridation, royalty, women judges and nudist camps; he learns them from his culture. But his genes may influence which ones he elects to put on his tray. Different cultural institutions – family, church, school, books, television – like different cafeterias, serve up somewhat different menus, and the choices a person makes will reflect those offered him as well as his own biases. As he gets older, choice of cafeterias will become important, in addition to his choice of dishes within them” (Loehlin 1997, p.48).
